# Frontal Lobe Morphometry with MRI in a Normal Age Group of 6-17 Year-Olds

**DOI:** 10.5812/iranjradiol.10044

**Published:** 2012-12-27

**Authors:** M İlkay Koşar, İlhan Otağ, Vedat Sabancıoğulları, Mehmet Atalar, Hasan Tetiker, Aynur Otağ, Mehmet Çimen

**Affiliations:** 1 Department of Anatomy, Faculty of Medicine, Cumhuriyet Üniversity, Sivas, Turkey; 2 Vocational School of Health Services, Cumhuriyet Üniversity, Sivas, Turkey; 3 Department of Radiology, Faculty of Medicine, Cumhuriyet Üniversity, Sivas, Turkey; 4 Department of Anatomy, Faculty of Medicine, Muğla Üniversity, Muğla, Turkey; 5 Physical Education and Sport High School, Cumhuriyet Üniversity, Sivas, Turkey

**Keywords:** Frontal Lobe, Child, Magnetic Resonance Imaging

## Abstract

**Background:**

Morphometric data of the frontal lobe are important for surgical planning of lesions in the frontal lobe and its surroundings. Magnetic resonance imaging (MRI) techniques provide suitable data for this purpose.

**Objectives:**

In our study, the morphometric data of mid-sagittal MRI of the frontal lobe in certain age and gender groups of children have been presented.

**Patients and Methods:**

In a normal age group of 6-17-year-old participants, the length of the line passing through predetermined different points, including the frontal pole (FP), commissura anterior (AC), commissura posterior (PC), the outermost point of corpus callosum genu (AGCC), the innermost point of corpus callosum genu (IGCC), tuberculum sella (TS), AGCC and IGCC points parallel to AC-PC line and the point such line crosses at the frontal lobe surface (FCS) were measured in three age groups (6-9, 10-13 and 14-17 years) for each gender.

**Results:**

The frontal lobe morphometric data were higher in males than females. Frontal lobe measurements peak at the age group of 10-13 in the male and at the age group of 6-13 in the female. In boys, the length of FP-AC increases 4.1% in the 10-13 age group compared with the 6-9-year-old group, while this increase is 2.3% in girls.

**Conclusion:**

Differences in age and gender groups were determined. While the length of AGCC-IGCC increases 10.4% in adults, in children aged 6-17, the length of AC-PC is 11.5% greater than adults. These data will contribute to the preliminary assessment for developing a surgical plan in fine interventions in the frontal lobe and its surroundings in children.

## 1. Background 

Advanced MRI techniques provide anatomic brain images allowing morphometric studies (1, 2). These techniques include functional methods (3-8) which allow determination of total or regional volume, zone (1, 3) or length (7, 8) and also changes in the blood oxygenation level that reflect variations in neural activity.

MRI studies are especially suitable for children, because no ionizing radiation is used (3). Additionally, MRI studies provide convenience for composing gender, age, patient or normal sample groups. Considering brain development, understanding the differences between the groups is important for interpreting clinical imaging studies (9, 10).

In brain microsurgical procedures, the target is to reach the intracranial lesions by gentle retraction without touching the brain structures to minimize the risk of damage and complications (7). Most intracranial lesions are removed successfully by several approaches; subfrontal through the lamina terminalis, transcallosal through the corpus callosum, transcortical-transventricular through the lateral ventricle, pterional, interhemispheric transcaollosal-interforniceal and transcallosal subchoroidal and combinations thereof (11). The subfrontal method is preferred for lesions situated completely above the diaphragma sella (7).

Normal brain morphometric data for the microsurgical operation plans have mostly been performed in adults. The pediatric brain is different from the adult brain in shape and size and continues to develop during childhood. In all head measurements, there is a strong association with age, which is mentioned frequently in the frontal area (12). As the white matter increases with age, the gray matter decreases (4, 9, 13). Frontal lobe is among the latest developing regions. Along with developmental and personal maturation, it is one of the most expanding cortical regions (14). It reaches the adult size hardly by 20 years old (2). Brain morphometry differs according to gender as well as age. Brain volume reaches the highest level at the age of 10.5 in girls and 14.5 in boys (4). The frontal gray matter peaks at the age of 11.0 in girls and 12.1 in boys (2). The brain volume is 7-10% greater in the male gender compared to the female gender (4, 5, 15).

During brain development, there is an age-related gender difference (4, 5, 12, 15). This study presents the effect of age and gender in pediatric brain development on the frontal lobe morphometry at mid-sagittal cross-section. These morphometric data will help surgical planning in pediatric subfrontal operations.

## 2. Objectives

Knowledge of these measurements in this study will allow exact planning of surgical approaches to the frontal lobe in certain age and gender groups of children.

## 3. Patients and Methods 

**Figure 1:**
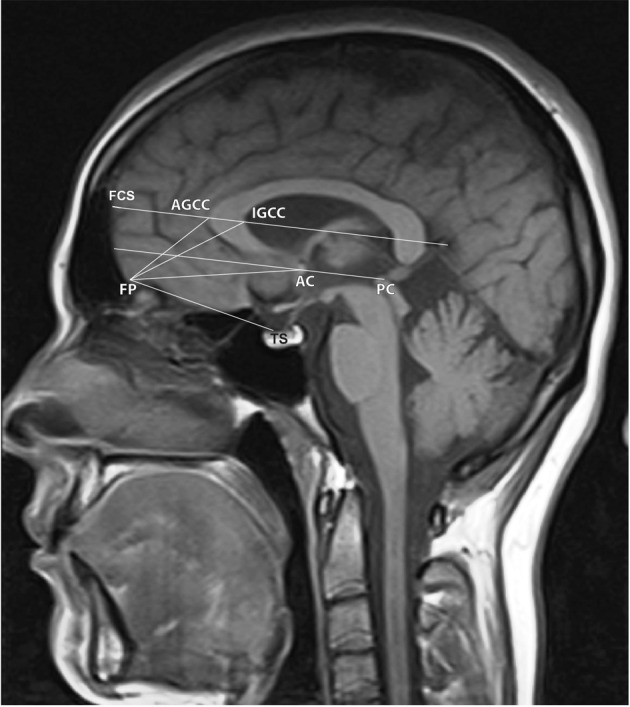
The midsagittal section of the brain MRI with schematic illustration of landmarks and reference lines; FP, frontal pole; AC, commissura anterior; PC, commissura posterior; AGCC, the outermost point of corpus callosum genu; IGCC, innermost point of corpus callosum genu; TS, tuberculum sella, AGCC and IGCC points parallel to the AC-PC line and the point such a line crosses at the frontal lobe surface (FCS).

Our study was carried out retrospectively after the approval of the local ethics committee on MRIs of 90 brains with normal structures aged 6 to 17 years living in the city of Sivas, Turkey. Only subjects who met all the following criteria entered the study: 1) subjects with no previous brain disease; 2) no signal abnormality and cerebral tumors, infarction or hemorrhage on MRI; 3) no positive trauma history; 4) no significant medical, neurological or psychiatric disorder or history of head injury or loss of consciousness; 5) no history of prenatal confounds that may influence brain development, such as prenatal exposure to substances or obstetrical complications. The subjects were divided into three age groups as 6-9, 10-13 and 14-17. Each age group was composed of 30 subjects as 15 girls and 15 boys. The research was performed on a Model 2001 1.5 Tesla MRI device (Exelart, Toshiba, Tokyo, Japan) in the Department of Radiology, Research and Training Hospital, Faculty of Medicine, Cumhuriyet University. The images were taken by sagittal T1 weighted fast rapid spin echo (SE) technique; Repetition time (TR): 5000 ms; Echo time (TE): 1094 ms; Flip angle (FA): 90/16, Number of excitation (NEX):2; Field of view (FOV): 180 × 220 mm; Matrix size: 224 × 320; Section Slice Thickness: 6.2 mm. Evaluation of images were carried out using Toshiba software (V4.10, Excelart, Tokyo, Japan). Screen resolution was 1024 × 768 pixels. All images were evaluated by an observer with 11-years of experience in pediatric neuroradiology interpretation. Over midsagittal view images; the distances between frontal pole-commissura anterior (FP-AC), frontal pole-commissura posterior (FP-PC), frontal pole-outmost point of corpus callosum genu (FP-AGCC), frontal pole-innermost point of corpus callosum genu (FP-IGCC), frontal pole- tuberculum sella (FP-TS), commissura anterior-commissura posterior (AC-PC), the point that the line passing through AGCC and IGCC points parallel to AC-PC crosses at the frontal lobe surface–outermost point of corpus callosum genu (FCS-AGCC), the point that the line passing through AGCC and IGCC points parallel to AC-PC crosses at the frontal lobe surface-commissura anterior (FCS-AC) and the outermost point of corpus callosum genu–innermost point of corpus callosum genu (AGCC-IGCC) were measured (Figure 1). The measurements on magnetic resonance imaging were carried out by electronic measurement techniques available on MR console. All measurements of FP-AC, FP-PC, FP-AGCC, FP-IGCC, FP-TS, AC-PC, FCS-AGCC, FCS-AC and AGCC-IGCC values have been presented as mean ± SD.

## 4. Results

In all participants, the mean FP-AC length was 56.8 mm (58.1 mm in males, 55.7 mm in females), the mean FP-AGCC length was 38.0 mm (38.4 mm and 37.7 mm, respectively), the mean FP-IGCC length was 46.0 mm (46.5 mm and 45.5, respectively), the mean FP-TS length was 47.4 mm (48.5 mm and 46.3 mm, respectively). In addition, the mean FCS-AGCC length was 35.5 (36.1 mm and 34.9 mm, respectively), the mean FP-AGCC length was 38.0 mm (38.4 mm and 37.7 mm, respectively), the mean FCS-AC length was 63.0 mm (63.7 mm and 62.4 mm, respectively), the mean AGCC-IGCC length was10.8 mm (10.7 mm and 11.0 mm, respectively) and the mean AC-PC length was 24.7 mm (24.6 mm and 24.7 mm, respectively) (Table 1).

**Table 1:** Frontal Lobe Morphometric Measurements in the 6 to 17 (y) Age Group

	6-17 Age (y)					
	**Total (n = 90 )**		**Boys (n = 45)**		**Girls (n = 45)**	
	Mean±SD (mm)	Range (mm)	Mean±SD (mm)	Range (mm)	Mean±SD (mm)	Range (mm)
**FP-AC**	56.8±4.7	38.0-65.0	58.1±4.3	43.0-65.0	55.7±4.7	38.0-63.0
**FP-AGCC**	38.0±2.8	31.0-45.0	38.4±3.0	32.0-45.0	37.7±2.6	31.0-43.0
**FP-IGCC**	46.0±3.0	37.0-55.0	46.5±3.3	37.0-55.0	45.5±2.7	39.0-51.0
**FP-TS**	47.4±4.7	33.0-59.0	48.5±4.5	38.0-58.0	46.3±4.7	33.0-59.0
**FCS-AGCC**	35.5±3.4	28.0-54.0	36.1±4.0	30.0-54.0	34.9±2.7	28.0-41.0
**FCS-AC**	63.0±4.2	49.0-72.0	63.7±4.5	49.0-72.0	62.4±3.9	51.0-70.0
**AGCC-IGCC**	10.8±1.6	7.0-15.0	10.7±1.8	7.0-15.0	11.0±1.5	8.0-15.0
**AC-PC**	24.7±1.8	20.0-28.0	24.6±1.9	20.0-28.0	24.7±1.7	20.0-28.0

After dividing all participants into three 6-9, 10-13 and 14-17-year-old groups; morphometric measurements of the frontal lobe (except for FP-AGCC) show the fastest development in the 10-13-year-old boys while this development is slower in girls. The FP-AGCC distance decreased with age in both genders and it was also smaller in girls in all age groups (Tables 2, 3 and 4).

**Table 2:** Frontal Lobe Morphometric Measurements in the 6 to 9 (y) Age Group

	6-9 Age (y) (n = 30)			
	**Boys (n = 15)**		**Girls (n = 15)**	
	Mean±SD (mm)	Range (mm)	Mean±SD (mm)	Range (mm)
**FP-AC**	57.2±5.4	43.0-63.0	54.8±6.4	38.0-63.0
**FP-AGCC**	39.4±2.9	32.0-43.0	38.2±2.3	34.0-42.0
**FP-IGCC**	46.5±4.1	37.0-55.0	45.4±2.9	41.0-49.0
**FP-TS**	48.0±5.7	38.0-58.0	47.1±5.4	35.0-59.0
**FCS-AGCC**	36.4±3.3	30.0-44.0	35.6±2.7	32.0-41.0
**FCS-AC**	63.4±5.5	49.0-72.0	62.2±2.5	58.0-66.0
**AGCC-IGCC**	10.5±1.9	7.0-14.0	10.5±1.1	8.0-12.0
**AC-PC**	24.2±1.9	21.0-27.0	24.2±1.4	22.0-27.0

**Table 3:** Frontal Lobe Morphometric Measurements in the 10 to 13 (y) Age Group

	10-13 Age (y) (n = 30)			
	**Boys (n = 15)**		**Girls (n = 15)**	
	Mean±SD (mm)	Range (mm)	Mean±SD (mm)	Range (mm)
**FP-AC**	59.6±4.1	50.0-65.0	56.1±4.0	46.0-62.0
**FP-AGCC**	38.1±2.9	34.0-45.0	37.8±2.8	31.0-43.0
**FP-IGCC**	47.4±2.9	41.0-54.0	45.3±2.7	39.0-51.0
**FP-TS**	49.2±4.1	43.0-57.0	45.6±5.0	33.0-53.0
**FCS-AGCC**	37.2±5.7	32.0-54.0	34.5±2.8	31.0-40.0
**FCS-AC**	64.6±4.2	56.0-71.0	61.5±5.0	51.0-68.0
**AGCC-IGCC**	11.0±1.6	9.0-15.0	10.6±1.4	8.0-13.0
**AC-PC**	25.0±1.7	22.0-28.0	24.2±1.8	20.0-27.0

**Table 4:** Frontal Lobe Morphometric Measurements in the 14 to 17 (y) Age Group

	14-17 Age (y) (n = 30)			
	**Boys (n = 15)**		**Girls (n = 15)**	
	Mean±SD (mm)	Range (mm)	Mean±SD (mm)	Range (mm)
**FP-AC**	57.7±2.8	52.0-62.0	56.0±3.6	49.0-61.0
**FP-AGCC**	37.5±3.0	33.0-42.0	37.3±2.6	32.0-42.0
**FP-IGCC**	45.4±2.7	40.0-49.0	45.9±2.8	40.0-50.0
**FP-TS**	48.4±3.7	41.0-54.0	46.2±3.8	40.0-54.0
**FCS-AGCC**	34.7±1.9	31.0-37.0	34.7±2.5	28.0-39.0
**FCS-AC**	63.3±3.8	55.0-68.0	63.4±3.7	56.0-70.0
**AGCC-IGCC**	10.6±1.9	7.0-14.0	11.7±1.6	10.0-15.0
**AC-PC**	24.6±2.1	20.0-28.0	25.7±1.6	23.0-28.0

All the above mentioned measurements were obtained in the three age groups for both genders (Tables 2, 3 and 4).

## 5. Discussion

We studied the frontal lobe morphometry in normal 6 to 17-year-old children and the effect of age and gender on this morphometry. The frontal lobe is the largest of the four main lobes. There are many approaches for the treatment of lesions situated in the frontal lobe and its surrounding. MRI images provide anatomic brain data allowing brain morphometry studies during the development (2). MRI studies are especially suitable for children, because ionizing radiation is not used (4). We found that frontal lobe morphometric data obtained at mid-sagittal sections were higher in males. In the studies manifesting the difference of brain development according to genders, it was determined that the male brain volume is 7-10% greater than the female brain volume (4, 5, 15). The pattern of a greater brain volume in the male gender was also seen in children in the current study.

In all head measurements, there is a strong association with age, which is mentioned frequently in the frontal area (12). The frontal lobe enlarges gradually until the age of 8 and quickly grows between the ages of 8 and 14 (16). The brain volume reaches the highest level at the age of 10.5 in girls and 14.5 in boys (4). In our study, frontal lobe morphometric measurements (except for FP-AGCC) increase in boys at the age group of 10-13 and decreases at the age group of 14-17 and in girls,, this decrease is observed in many morphometric measurements at the age group of 10-13. The frontal lobe is among the latest developing regions. It reaches the adult size hardly at 20 years old (2, 17). Rapid development of the frontal lobe starts and ends sooner in the female (5). The range of morphometric measurements in the age group of 6-9 in the frontal lobe is wider in boys. In the other age groups, this range is similar between the genders. Frontal lobe is one of the brain regions that expands most along with development and maturation (14). As well as the difference in brain development between age groups, there is also developmental differences between genders (9, 13, 17).

This study presenting differences in frontal lobe morphometry between age and gender groups in normal 6 to 17-year-old children will guide to the plans that should be applied in sensitive surgical operations which will be performed through the subfrontal approach preferred for lesions situated completely above the diaphragma sella, including the pituitary adenoma, craniopharyngioma, clival chordoma, meningioma originated from tuberculum sella, hypothalamus and optic nerve glioma, dermoid cyst, teratoma and third ventricle tumors. When the entire head is taken into consideration, age has a powerful impact on all dimensions. This impact is more applied to the frontal lobe (12). The frontal lobe grows fast from 8 to 14 years of age (16). Fast development of the frontal lobe starts and ends earlier in girls (5). In our study, morphometric measurements of the frontal lobe (except for FP-AGCC) show the fastest development in the 10-13 age group in boys, while this development is slower in girls. In boys, the length of FP-AC increases 4.1% in the 10-13 age group compared with the 6-9-year-old group, while this increase is 2.3% in girls. The length of FP-AGCC decreases in successive age groups of 6-9, 10-13 and 14-17 in both genders. When this measurement is compared with the study carried out on adult brains, it is seen that this decrease continues (7). The length of FP-AC is 7.6% greater in adults than children aged 6-9 regardless of gender. While this difference is 5% according to the 10-13 years age group, it is 6.1% greater in children aged between 14 and 17. Comparing our results with the study conducted by Ardeshiri et al. (7), the measurements carried out in the adult brain are higher (Table 5). This data supports studies implying that development of the frontal lobe continues throughout life (17, 18). The reason for the decrease in the distance between the points on the frontal surface of the frontal lobe (FP and FCS) and corpus callosum (AGCC) with aging is that corpus callosum comes close to the anterior part of the frontal lobe as it develops faster than the frontal lobe (2). While the length of AGCC-IGCC increases 10.4% in adults, in children aged 6-17, the length of AC-PC is 11.5% greater than adults.

**Table 5:** Morphometric Measurements and Differences of the Frontal Lobe in Adults and Children Aged 6 to 17 (y)

Distance (mm)	Our study (6-17 age)	Ardeshiri et al. (Adult)	Increases (%)
**FP-AC**	57.2	60.3	5.4
**FP-AGCC**	39.4	37.1	-5.8
**FP-IGCC**	46.5	47.2	1.5
**FP-TS**	48.0	48.8	1.6
**FCS-AGCC**	36.4	36.0	-1.0
**FCS-AC**	63.4	65.3	2.9
**AGCC-IGCC**	10.5	11.6	10.4
**AC-PC**	24.2	27.0	11.5

Developmental differences between genders in addition to age groups are observed in brain development (13, 18). The present study will help plan sensitive surgical operations by the sub-frontal approach, which may be applied to tuberculum sella, third ventricle and its surroundings in children.
